# Effects of perceived variety-support on middle school students’ learning engagement in physical education: the mediating role of motivation

**DOI:** 10.3389/fpsyg.2024.1459362

**Published:** 2024-09-16

**Authors:** Miao Miao, Hongqin Chai, Rui Xue, Qi Wang

**Affiliations:** ^1^Department of Basic Courses, Shanxi Youth Vocational College, Taiyuan, China; ^2^School of Education, Beijing Sport University, Beijing, China; ^3^College of P.E. and Sports, Beijing Normal University, Beijing, China

**Keywords:** perceived variety-support, learning engagement, motivation, physical education, middle school students

## Abstract

**Introduction:**

High engagement in physical education (PE) could effectively develop students’ motor competence and promote physical activity, which was significantly important for students’ physical and mental health. Researches had shown that motivation was an important factor in explaining students’ learning engagement, and variety-support as the fourth independent psychological need was a potential factor influencing students’ learning motivation. However, there was a lack of empirical research evidence on the effect of perceived variety-support on middle school students’ learning engagement in PE and the influencing mechanisms. This study aimed to investigate the direct effect of perceived variety-support on learning engagement in PE and the mediating effect of motivation in PE on the relationship.

**Methods:**

A cross-sectional study was conducted and 587 middle school students from Liaoning province filled the paper-and-pencil questionnaires adopting perceived variety-support in PE scale (PVSPES), utrecht work engagement scale-student (UWES-S), and perceived locus of causality in PE scale, which had been proved to have good reliability and validity (294 boys and 293 girls, Mage=13.47 ± 0.94).

**Results:**

The results showed three variables were significantly positively correlated with each other (*r* = 0.323-0.562 *p* < 0.01) and perceived variety-support in PE could not only directly promote middle school students’ learning engagement in PE but also indirectly through the mediating effect of motivation in PE.

**Discussion:**

Therefore, in order to better promote students’ participation in PE class, we should pay more attention to satisfy students’ varied PE learning needs and stimulate students’ autonomous learning motivation.

## 1 Introduction

The advancement of digital technology and artificial intelligence has undoubtedly increased convenience but also led to an increase in screen exposure. This, in turn, has caused sedentary behaviors and resulted in a lack of physical activity (Nakshine et al., 2022).

A pooled analysis of 298 school-based surveys, encompassing 1.6 million participants aged 11–17 years, showed that 81% of school-going students in this age group were insufficiently physically active, indicating that the vast majority of these students are not meeting the current physical activity guidelines (Guthold et al., 2020).

Maintaining a physically active lifestyle during school years is critical for their overall health and mental well-being, including reduced sedentary behavior, improved scoliosis outcomes, enhanced cardiorespiratory and muscular fitness, healthy weight management, and positive effects on psychological health (Almahmoud et al., 2023; Jiang et al., 2023; Martinko et al., 2023). Physical education (PE) could be regarded as a powerful influence in promoting school-aged students’ physical activity (Wallhead and Buckworth, 2004).

As an indispensable school subject with course goals defined by curriculum standards, schools and PE teachers in China have been encouraged to include “health aims” into the physical and health education curriculum. These “health aims” focus on enhancing students’ physical and mental health and helping them adapt effectively to their external environment (Ji, 2022). Similarly, many countries have paid attention to reforming their physical and health education curricula to include these “health aims” (OECD, 2019). One of the key strategies for achieving these “health aims” is promoting physical activity through PE. Recent global evidence indicates that comprehensive school-based physical activity programs, especially those integrated into the PE curriculum, are more successful in promoting physical activity. Additionally, a supportive school environment is a crucial component of the adolescent physical activity system (van Sluijs et al., 2021). Therefore, greater emphasis needs to be placed on the PE curriculum in schools.

There was a global consensus that PE is the most important subject for students to learn motor skills, acquire communication and cooperation skills, and develop excellent personalities and values (McLennan and Thompson, 2015).

High-quality PE promoted motor literacy, equipped students with the motor skills necessary for lifelong physical activities (Whitehead, 2013), and provided potential evidence of a significant increase in physical activity levels among school-going students (Ha et al., 2019). The two main focal points of PE were PE teachers and students, and the attainment of high-quality PE inevitably predicated students’ learning engagement in PE lessons (Fredricks et al., 2004). Learning engagement is a positive, fulfilling state of mind related to learning, characterized by vigor, dedication, and absorption. It also reflects the degree of students’ efforts in learning, understanding, and mastering knowledge and skills (Schaufeli et al., 2002b). Vigor refers to the state in which students are fully energized in the learning process, willing to put in effort, and highly involved in learning; dedication refers to the state in which students have full enthusiasm for learning and are fully devoted to learning; and absorption refers to the state in which students concentrate on learning and enjoy the fun of learning (Ni and Wu, 2011). In the present middle school PE classroom teaching, due to the absence of attention to students’ subjective consciousness, the lack of emotional interaction between students and teachers, as well as among students, the low novelty and challenge of teaching content, tedious teaching methods and means, and other factors, students have low energy and enthusiasm in PE classes and show a low level of learning engagement (Zhang et al., 2020).

Low levels of learning engagement in PE directly lead to reduced exercise intensity and insufficient physical activity, which, over time, contribute to physical inactivity and negatively impact physical and mental health. Therefore, there is an urgent need to explore effective operational programs to improve students’ learning engagement in PE.

Improving students’ motivation for PE learning, especially intrinsic motivation, could inspire students to learn and improve their focus on learning. Research has shown that learning engagement is highly correlated with intrinsic motivation (Kuo et al., 2020; An et al., 2022; Alemayehu and Chen, 2023). According to self-determination theory (SDT), an individual’s participation in an activity may be motivated by relative self-determination or autonomy, and/or by more controlled motivation (Ryan et al., 2009), which indicates that stimulating students’ motivation in PE had positive integrative benefits for enhancing students’ learning engagement behaviors such as increased vigor, increased emotional engagement, and improved concentration. In addition, previous studies has indicated that when teaching and learning elements align with the psychological needs of students for autonomy, competence, and relatedness, students’ motivation, achievement, and well-being will be significantly enhanced, and it may have a positive impact on students’ learning.

The provision of variety-support was put forward as a potential influencing factor of one’s motivation in PE, the effects of which on physical activity had been proven to be positive, and increasing the variety-support provided for PE could increase an individual’s motivation of sport participation and enjoyment of sport (Juvancic-Heltzel et al., 2013). It has been reported that variety-support was the provided activity or environmental elements of variety with given contexts. Moreover, variety-support or non-support would probably facilitate (or hinder) subsequent variety experiences (Eather et al., 2022). In PE, variety-support was comprised of the provision of diverse teaching methods, sports equipment, curriculum contents, lesson topics, venues for classes, etc. Variety-support could maximize the positive impact of exercise on health and well-being by improving emotional reactions in sports contexts (Mandolesi et al., 2018). In addition, it has been pointed out that variety-support perceived by students in PE could influence their physical activity level (Sylvester et al., 2016b), predict physical activity behaviors (Sylvester et al., 2014a), and increase indices of exercise-related well-being (Sylvester et al., 2016a).

SDT emphasizes that individuals are sufficiently intrinsically motivated to participate in physical activity when three basic psychological needs (the need for competence, the need for autonomy, and the need for relatedness) are fulfilled (Teixeira et al., 2012). Sylvester et al. (2014b) extended this theory by suggesting that variety-support could be considered a fourth independent basic psychological need that influences motivation to participate in physical activity. Similarly, Abós et al. (2021) found that perceived task diversity predicted adolescents’ autonomous motivation in PE classes (Abós et al., 2021). Therefore, further research is needed to better understand students’ PE learning engagement and provide the rationale for new initiatives in students’ PE learning.

Perceived variety-support is currently considered an independent element that promotes motivation and behavior in physical activity; however, empirical research on this topic is relatively limited.

Moreover, studying middle school students’ learning engagement in PE is crucial for improving both their physical literacy and PE core literacy, and research on the middle school students’ learning engagement in PE also lacked new conceptual support. Consequently, this study aimed (1) to determine the relationships between perceived variety-support, motivation, and learning engagement in school PE settings and (2) to investigate the possible mediating effect of motivation in PE on the relation between perceived variety-support and learning engagement in PE among middle school students, in an effort to provide additional support for promoting middle school students’ learning in PE, advancing the achievement of high-quality PE, and increasing the physical activity levels of adolescents.

## 2 Methods

### 2.1 Participants and procedures

A convenience sample of 611 students from a middle school in Fuxin Province was used for this study, where mandatory PE classes were offered three times a week, each lasting 40 min throughout the school year. The PE classes in this study mainly referred to the junior high school PE and health curriculum in China’s compulsory education stage. To ensure voluntariness and confidentiality of participants in the study, students and their parents were required to provide written informed consent. The cross-sectional data for this study were collected over approximately 2 weeks in October 2023. With the assistance of the head teacher, students completed paper-and-pencil questionnaires during their self-study class. After excluding invalid questionnaires—those with missing information, inconsistent answers, or significant deviations from typical responses—we analyzed responses from 587 students, yielding a valid response rate of 96.1%. Among the respondents, there were 294 boys (50.1%) and 293 girls (49.9%), with 242 (41.2%) students in the first year, 153 (26.1%) in the second year, and 192 (32.7%) in the third year of junior high school. The age range of the students was from 12 to 16 years (Mage = 13.47 ± 0.94).

### 2.2 Instruments

#### 2.2.1 Perceived variety-support in PE

The Perceived Variety-Support in PE Scale (PVSPES) was developed and evaluated by Eather et al. (2022) to assess individual variety-support in PE, which had been shown to have good internal consistency, factorial validity, and test–retest reliability and to be suitable for use with adolescents (Eather et al., 2022). To ensure the content validity of the scale, first, two PE scholars majoring in PE Curriculum and Pedagogy were invited to translate the scale into Chinese, followed by proofreading, discussion, and integration. Second, two more PhD students majoring in English were invited to translate the Chinese version of the scale back into English and then compare it with the original scale accordingly. Finally, the four discussed the Chinese version of the scale together and finalized it. The final revised Chinese version was piloted before the formal study and proved to be adaptable. The scale included eight items and was a brief unidimensional scale measuring variety-support in PE activity, instruction, and environment (e.g., “In PE, my teacher provides a range of different sports equipment for me to use the term, e.g., bats, balls, hoops, racquets, nets”). Each question was scored on a 4-point Likert scale, ranging from 1 = never to 4 = always. The higher the score, the higher the level of the student’s perceived variety-support in PE. In the present study, the scale had good reliability (Cronbach’s *α* = 0.850) and ideal validity (χ2/df = 2.481, GFI = 0.982, AGFI = 0.963, NFI = 0.972, IFI = 0.983, RMSEA = 0.050).

#### 2.2.2 Learning engagement in PE

The Utrecht Work Engagement Scale-Student (UWES-S), originally developed by Schaufeli et al. (2002a) and later revised and sinicized by Fang et al. (2008), was adopted to measure middle school students’ learning engagement in PE. Combined with the characteristics of PE teaching and learning in middle school, “study” was replaced by “study in PE lessons.” For example, “When studying, I feel strong and vigorous” was replaced by “When studying in PE lessons, I feel strong and vigorous.” The 17-item scale was divided into three dimensions, including vigor (6 items, e.g., “I can continue for a very long time when I am studying in PE lessons”), dedication (5 items, e.g., “I find my studies in PE lessons to be full of meaning and purpose”), and absorption (6 items, e.g., “Time flies when I’m studying in PE lessons”). A 5-point Likert scale was used to measure the degree of students’ learning engagement in PE, ranging from 1 = strongly disagree to 5 = strongly agree. Higher scores indicated higher levels of students’ learning engagement in PE.

In this study, the Cronbach’s *α* for each dimension was as follows: 0.901 for vigor, 0.923 for dedication, and 0.884 for absorption, and the Cronbach’s α for the whole scale was 0.956, which showed good reliability. A confirmatory factor analysis was conducted and showed that the structural validity of the scale was adequate (χ^2^/df = 2.883, GFI = 0.938, AGFI = 0.911, NFI = 0.961, IFI = 0.974, RMSEA = 0.057).

#### 2.2.3 Motivation in PE

The degree of middle school students’ self-determined motivation in PE was assessed by adopting the Revised Perceived Locus of Causality in PE Scale (PLOC-R) revised by Vlachopoulos et al. (2011). The scale can indicate the extent to which middle school students choose to take part in PE for different purposes. In accordance with the same translation and revision process as for PVSPES, this study finalized the Chinese version of the PLOC-R, which includes nineteen items. The scale consisted of five factors: motivation (four items, e.g., “I take part in PE, but I really do not know why”), external regulation (three items, e.g., “I take part in PE because in this way I will not get a low grade”), introjected regulation (four items, e.g., “I take part in PE because I would feel bad if the teacher thought that I am not good at PE”), identified regulation (four items, e.g., “I take part in PE because it is important to me to do well in PE”), and intrinsic motivation (four items, e.g., “I take part in PE because PE is enjoyable”). The subjects responded using a 7-point Likert scale, with 1 indicating strongly disagree, 7 signifying strongly agree, and 4 serving as the neutral midpoint. A Relative Autonomy Index (RAI) was formed as an indication of individual differences in the degree of self-determination of his/her behavior. The RAI score was obtained by multiplying each item score by a given weight: motivation was given a weight of −3, external regulation −2, introjected regulation −1, identified regulation +1, and intrinsic motivation +2. Then, the sum of all products ranged from −39 to 15, with a higher RAI score signifying more self-determined motivation. In the present study, the Cronbach’s *α* of five dimensions was as follows: 0.907 for motivation, 0.855 for external regulation, 0.874 for introjected regulation, 0.820 for identified regulation, and 0.956 for intrinsic motivation. The Cronbach’s α of the whole scale was 0.830, representing good internal consistency. Meanwhile, the scale had good validity (χ2/df = 2.902, GFI = 0.933, AGFI = 0.906, NFI = 0.956, IFI = 0.971, RMSEA = 0.057) in the present study.

### 2.3 Data analysis

Before statistical analysis, the common method deviation test and normal distribution test were conducted using SPSS 26.0. In this study, data were collected by filling out paper-and-pencil questionnaires, and several scales were used simultaneously. There were differences in subjects’ grades and classes. Therefore, common method deviation required attention. To control for potential common method deviation, the Harman single-factor analysis was conducted to test the possibility of systematic errors. The results showed that, in the principal component analysis without varimax rotation, there were six factors with eigenvalues greater than 1. The variance explanation rate of the first factor without rotation was 32.576%, which is less than the 40% critical value, indicating that the common method deviation in this study was within acceptable limits. Moreover, all variables were tested to fit a normal distribution.

SPSS 26.0 was employed to conduct a reliability analysis of three scales, presenting Cronbach’s *α* for each scale, as well as descriptive statistics including mean scores and standard deviations (SD) for the three variables. Additionally, independent-samples T-tests and one-way analyses of variance were performed alongside Pearson’s correlation analysis. Amos 26.0 was utilized to carry out a confirmatory factor analysis (CFA) to assess the factorial validity of the three scales. The mediating effect serves to analyze the influence process and mechanisms through which independent variables affect dependent variables, establishing itself as an essential statistical method for examining relationships among multiple variables (Wen et al., 2022). In this study, the SPSS 26.0 PROCESS macro was applied to evaluate the significance of the mediating effect, while Bootstrap methods were employed to calculate mediation effect sizes.

## 3 Results

### 3.1 Descriptive characteristics

Table 1 presents the descriptive characteristics of 587 samples and the differences in gender and grade for each variable. The mean scores for perceived variety-support in PE, learning engagement in PE, and motivation in PE were 2.807 (SD = 0.703), 3.650 (SD = 0.832), and 0.938 (SD = 9.411), respectively. There were no gender differences in perceived variety-support in PE and motivation in PE; however, boys showed high levels of learning engagement in PE (*p* < 0.001). Additionally, there were no significant grade differences in learning engagement or motivation in PE, although first-year students reported lower levels of perceived variety-support compared to second- and third-year students (*p* < 0.001).

**Table 1:** Descriptive characteristics of the sample.

Gender		*t*	*p*	Grade		
Boys (*n* = 294)	Girls (*n* = 293)			Junior 1 (*n* = 242)	Junior 2 (*n* = 153)	Junior 3 (*n* = 192)
2.831 (0.728)	2.782 (0.677)	0.841	0.401	2.548 (0.679)	3.022 (0.693)	2.962 (0.636)
3.775 (0.891)	3.525 (0.748)	3.682	**<0.001**	3.621 (0.810)	3.753 (0.840)	3.603 (0.850)
1.432 (10.003)	0.443 (8.767)	1.273	0.203	1.562 (8.729)	1.040 (10.088)	0.072 (9.662)

A p-value of less than 0.001 signifies a statistically significant difference.

### 3.2 Correlations between research variables

Table 2 presents the results of Pearson’s correlation analysis between research variables. Perceived variety-support in PE was significantly positively correlated with learning engagement in PE (*r* = 0.408, *p* < 0.01) and motivation in PE (*r* = 0.323, *p* < 0.01). Learning engagement in PE was significantly positively correlated with motivation in PE (*r* = 0.562, *p* < 0.01).

**Table 2:** Correlations between research variables.

Variables	1	2	3
1. Perceived variety-support in PE	1		
2. Learning engagement in PE	0.408^**^	1	
3. Motivation in PE	0.323^**^	0.562^**^	1

**p < 0.01.

### 3.3 Mediating effect analysis

**Figure 1:**
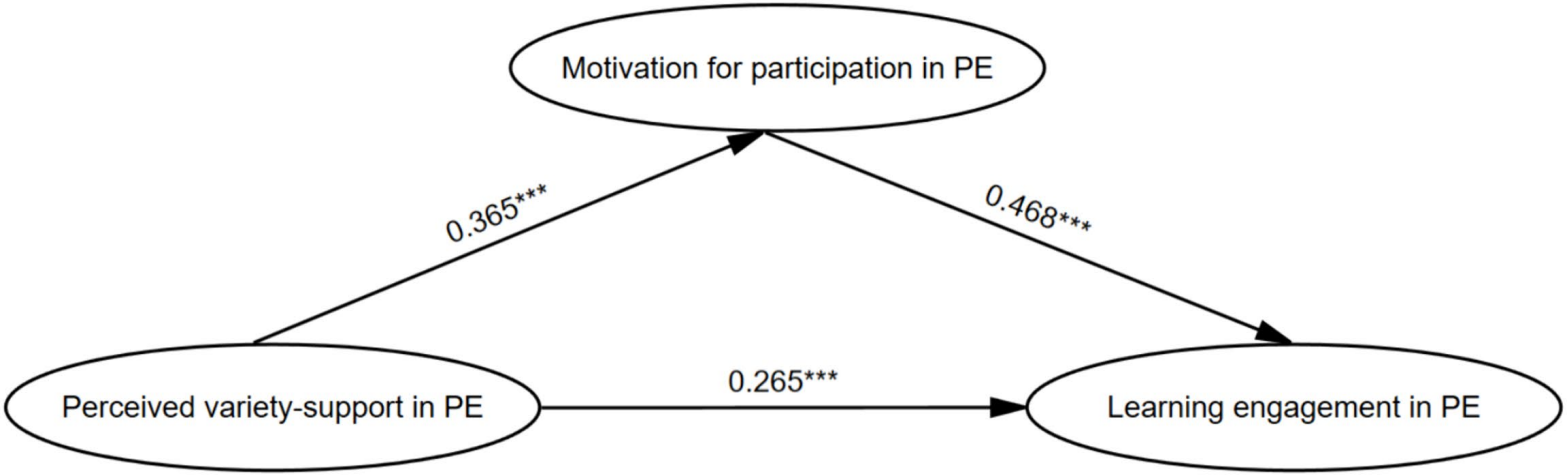
Path model of perceived variety-support in PE affecting learning engagement in PE.

Table 3 displays the results of the mediating effect test. The results showed that the positive predictive effect of perceived variety-support in PE on learning engagement in PE was significant (standardized path coefficient = 0.435, SE = 0.038, *p* < 0.001). Even after adding motivation in PE as a mediating variable, the direct positive effect of perceived variety-support on learning engagement in PE remained significant (standardized path coefficient = 0.265, SE = 0.036, *p* < 0.001). Perceived variety-support in PE had a significant positive predictive effect on motivation in PE (standardized path coefficient = 0.365, SE = 0.040, *p* < 0.001), and motivation in PE had a significant positive predictive effect on learning engagement in PE (standardized path coefficient = 0.468, SE = 0.035, *p* < 0.001). In addition, the total effect of perceived variety-support in PE on learning engagement in PE was 0.435 (Bootstrap 95%CI = [0.351, 0.521]). The direct effect was 0.265 (Bootstrap 95%CI = [0.184, 0.348]), accounting for 60.92% of the total effect. The mediating effect of motivation in PE was 0.170 (Bootstrap 95%CI = [0.123, 0.225]), accounting for 39.08% of the total effect. The 95% confidence interval of Bootstrap did not contain 0 (see Table 4). Therefore, middle school students’ perceived variety-support in PE could positively affect learning engagement in PE, but middle school students’ motivation in PE also had a mediating effect on perceived variety-support in PE and learning engagement in PE (see Figure 1).

**Table 3:** Path coefficients of the mediation model.

IV	DV	M	Effect of IV on M		Effect of M *n* DV		Effect of IV on DV		Effect of IV on DV adding M	
			Coeff	SE	Coeff	SE	Coeff	SE	Coeff	SE
Perceived variety-support in PE	Learning engagement in PE	Motivation in PE	0.365***	0.040	0.468***	0.035	0.435***	0.038	0.265***	0.036

IV = independent variable; DV = dependent variable; M = mediator; ***p < 0.001.

**Table 4:** Values of total, direct, and indirect effects.

	Value	Proportion	95%CI	
			Lower	Upper
Total effect	0.435		0.351	0.521
Direct effect	0.265	60.92%	0.184	0.348
Indirect effect	0.170	39.08%	0.123	0.225

## 4 Discussion

The purposes of this study were (1) to examine the relationships between perceived variety-support, motivation, and learning engagement in PE among middle school students and (2) investigate the potential mediating effect of motivation in PE on the relationship between perceived variety-support and learning engagement. The results highlighted the significantly positive correlations between perceived variety-support, motivation, and learning engagement in PE and showed that perceived variety-support in PE could not only positively and directly predict learning engagement in PE but also positively and indirectly predict through the mediation of motivation in PE.

First, research has shown that middle school students’ engagement in PE is positively correlated with perceived variety-support in PE classes, which is consistent with the findings of previous studies (Martinek et al., 2019; Otundo and Garn, 2019). Students exhibited noticeable differences in their PE learning, reflecting a wide range of individual needs. The inherent disparities in motor competence among individuals, coupled with varying interests in sports, created a significant level of diversity in students’ PE learning abilities. The provision of variety-support by teachers in PE classes fostered student engagement, creating a conducive classroom environment and fostering harmonious teacher–student relationships. This, in turn, addressed the diverse learning needs of students (Furrer and Skinner, 2003). Otundo and Garn (2019) highlighted the predictive effects of situational interest and need-supportive teaching on students’ interest and engagement. This finding further demonstrated that when students’ learning attitudes and behaviors in PE were driven by their interests, their learning process became more autonomous, voluntary, comprehensive, and sustainable (Kirch et al., 2021). The introduction of variety in PE classes highlighted diverse motor competencies and movement skills, allowing students to experience a wide range of movements, games, and sports cultures. With careful planning, PE lessons offering a variety of sports equipment, locations, activities, curriculum goals, engaging teaching methods, and groupings can effectively cater to students’ diverse needs (Bertills et al., 2019). This approach generates interest in learning and sustainably motivates students to focus on PE learning in the classroom.

Second, the study confirmed that motivation in PE mediates the relationship between perceived variety-support and learning engagement. Sylvester et al. (2014a) found that perceived variety indirectly predicts physical activity behavior through autonomous motivation, aligning with our findings. Research indicated that variety, novelty, choice, and effort-based praise enhanced autonomous motivation toward PE (White et al., 2021), and motivation predicted intentional behavior participation (Ryan and Deci, 2017). The self-determination theory (SDT) revealed the extent to which and the manner in which various contextual factors influenced individuals’ exercise behavior, particularly through psychological experiences (Lange et al., 2011). Embedded within SDT, it is highlighted that people universally possessed innate basic psychological needs, and the degree to which these needs were satisfied within the social environment subsequently influenced individuals’ behavior, mediated by autonomous motivation. Specifically, autonomous and intrinsic motivation led to more adaptive outcomes in PE classes, including learning enjoyment and physical activity intentions (Vasconcellos et al., 2020), boosting student engagement. Therefore, variety-support in PE that satisfied students’ psychological needs sparked their intrinsic motivation to learn PE, further promoting engagement behaviors. This insight aided physical educators in designing high-quality PE sessions. Additionally, Tang and Ma (2023) emphasized that teaching content and learning/practicing modes shape students’ fundamental learning experiences in PE. The more the teaching content aligned with students’ learning needs, the more choices of sports equipment and venues were available, along with novel and interesting learning/practicing modes, the higher the student engagement in PE lessons (Tang and Ma, 2023). However, it is unrealistic to address all students’ needs simultaneously in terms of teaching content and facilities in PE classes. Thus, careful design of learning and practicing methods is crucial, as it facilitates the presentation, connection, and transition of knowledge and skills, intensifies learning challenges, maintains student motivation (Light, 2014), mitigates teaching–learning conflicts (Kinder et al., 2020), and ensures student engagement in PE lessons.

Finally, these findings has certain implications for school-based interventions. On the one hand, schools should implement high-quality PE to promote students’ participation in PE classes and foster a lifelong pursuit of a physically active lifestyle, which includes providing students with adequate opportunities for physical activity, conducting systematic professional training for PE teachers, being equipped with a complete evaluation and supervision system, and creating a dynamic PE atmosphere. On the other hand, PE teachers should provide students with variety-support in the PE curriculum and teaching to greatly satisfy the students’ needs for PE learning and promote active participation of students in PE classroom learning. For example, PE teachers should provide students with more space for independent practice, management, and choice to create an exploratory learning atmosphere, and they should also formulate more abundant and humanized teaching contents and learning goals according to individual differences (Zhang et al., 2020). It is also recommended to focus on selecting novel learning and practicing methods in pedagogical practice that stimulate students’ motivation and increase their interest in learning independently (Lentillon-Kaestner and Roure, 2019).

## 5 Conclusion

Studying how physical educators provide variety-support in PE and its impact on students’ motivation to learn PE lessons was of interest in understanding the poor performance of some middle school students in PE. This study concluded the direct effect of perceived variety-support on middle school students’ learning engagement in PE and the indirect motivation in PE on the relationship between perceived variety-support and learning engagement. On the one hand, gaining a better understanding of high learning engagement in PE was crucial to increase and guarantee the provision of variety-support in PE. On the other hand, high levels of autonomous and intrinsic motivation to learn PE made students perform more behaviors of learning engagement in PE lessons (Leo et al., 2022).

## 6 Limitations and implications for future research

This study provides empirical evidence to support the development of high-quality PE and the enhancement of students’ engagement in PE lessons. However, there are some limitations in this study. First, the present study depended on students’ ability to accurately reflect on their previous PE experiences to define the relationships between the variables. Alternative methods other than questionnaire surveys, which may better capture students’ perceptions of variety-support, motivation, and learning engagement in PE, should be considered. In future studies, interviews can be conducted to explore students’ interpretations of reconstructed descriptions of their school PE experiences, providing a deeper understanding of their engagement in PE class. Longitudinal studies are also recommended to analyze the development process of students’ perceived variety-support, motivation, and learning engagement in PE.

Second, PE classes can differ significantly across different countries, and this study only focused on the junior high school PE and health curriculum in China’s compulsory education stage. Future studies could include comparative experimental studies to explore the impact of perceived variety-support on students’ learning engagement across different curriculum modes, determining whether these effects are consistent across diverse educational contexts.

Furthermore, it would be important to determine whether this effect is consistent across different curriculum models. Finally, this study focused solely on the direct relationship between perceived variety-support, motivation, and learning engagement within the school PE context. However, there may be multiple mediating or moderating factors at play in practice. Future research should incorporate a broader range of variables to better understand the complex processes through which perceived variety-support affects learning engagement.

## Data Availability

The raw data supporting the conclusions of this article will be made available by the authors, without undue reservation.
